# Oscillatory Activity in Mouse Lemur Primary Motor Cortex During Natural Locomotor Behavior

**DOI:** 10.3389/fnsys.2021.655980

**Published:** 2021-06-18

**Authors:** Banty Tia, Fabien Pifferi

**Affiliations:** UMR 7179 CNRS/MNHN, Brunoy, France

**Keywords:** body posture, high β, locomotor cycle, low γ, *Microcebus murinus*

## Abstract

In arboreal environments, substrate orientation determines the biomechanical strategy for postural maintenance and locomotion. In this study, we investigated possible neuronal correlates of these mechanisms in an ancestral primate model, the gray mouse lemur. We conducted telemetric recordings of electrocorticographic activity in left primary motor cortex of two mouse lemurs moving on a branch-like small-diameter pole, fixed horizontally, or vertically. Analysis of cortical oscillations in high β (25–35 Hz) and low γ (35–50 Hz) bands showed stronger resting power on horizontal than vertical substrate, potentially illustrating sensorimotor processes for postural maintenance. Locomotion on horizontal substrate was associated with stronger event-related desynchronization than vertical substrate, which could relate to locomotor adjustments and/or derive from differences in baseline activity. Spectrograms of cortical activity showed modulation throughout individual locomotor cycles, with higher values in the first than second half cycle. However, substrate orientation did not significantly influence these variations. Overall, these results confirm that specific cortical mechanisms are solicited during arboreal locomotion, whereby mouse lemurs adjust cortical activity to substrate orientation during static posture and locomotion, and modulate this activity throughout locomotor cycles.

## Introduction

Studies investigating the neurophysiological mechanisms of locomotion concur to the idea that basic locomotor patterns are driven by spinal interneuronal networks, termed central pattern generators, which produce rhythmic activity in flexor/extensor muscles and achieve interlimb coordination (Takakusaki, 2013; Drew and Marigold, 2015; Kiehn, 2016). These spinal networks cooperate with descending signals from supraspinal centers hierarchically organized in the cerebrum, brainstem and cerebellum to trigger, adapt and stop the locomotor pattern (Rossignol et al., 2006; Gwin et al., 2011). The cerebral cortex is more specifically involved in the supervision of downstream circuits in situations requiring precise control and high levels of accuracy (Drew et al., 2002; Rossignol et al., 2006; Digiovanna et al., 2016). The motor cortex contains populations of pyramidal tract neurons which regulate the duration and level of activity of synergistic muscle groups throughout the gait cycle (Drew et al., 2008; Drew and Marigold, 2015).

Increased cortical involvement in the more complex locomotor tasks is particularly relevant to the current study. Here, we were interested in targeting cortical contribution to primate locomotion on substrates that mimic an arboreal environment. This question was rarely addressed in the literature since experimental paradigms generally focus on treadmill walking/stepping movements that allow better control of task parameters and more regular gait cycles (Gwin et al., 2011; Digiovanna et al., 2016). By contrast, research in the fields of biomechanics or ethology provided detailed study of various substrate types along with locomotor constraints and strategies (Schmitt, 2003b; Shapiro et al., 2016). We attempt to complement these aspects by investigating cortical mechanisms subtending these locomotor adaptations. Arboreal locomotion differs from the classic stepping task in that it involves coordinated fore- and hindlimb prehension (Schmitt, 2003b). Since grasping movements are controlled by a fronto-parietal cortical network in primates (Castiello, 2005; Tia et al., 2017), arboreal locomotion could more heavily rely on cortical circuits than stepping movements. To address this question, we focused on primary motor cortex oscillatory activity in high β (25–35 Hz) and low γ (35–50 Hz) ranges.

Authors suggest that the β band (≈15–35 Hz) is composed of multiple narrow bands with distinct locations and functional significance, including a high β (>20 Hz) and a low β (<20 Hz) band (Szurhaj et al., 2003; Kilavik et al., 2013). Sensorimotor β oscillations are typically characterized by a power decrease during movement preparation and execution, followed by a transient increase after movement end, and tonic increase during object grasping (Kilavik et al., 2013; Zaepffel et al., 2013). β power suppression during movement generally shows little specificity to task features, although it is somatotopically organized (Miller et al., 2007; Kilavik et al., 2013). As concerns the low γ band (30–50 Hz), studies are more heterogeneous regarding movement-related power variation (Szurhaj et al., 2003) and occasionally reported nonsignificant modulation (Omlor et al., 2007). Event-related desynchronization (ERD) typically occurs in low (<35 Hz) frequencies and event-related synchronization (ERS) in high (>50 Hz) frequencies, with rest and active spectra intersecting around 40–50 Hz (Miller et al., 2007). When low γ synchronization is measured, it is generally more discrete and somatotopically specific than β desynchronization (Crone et al., 1998). With regard to human locomotion, β oscillations (18–30 Hz) are suppressed in central sensorimotor areas during walking as compared to upright standing (Seeber et al., 2014, 2015). Cortical activity in β/low γ frequencies is coupled to gait cycle phase and strongest β power increase occurs around contralateral limb push-off, namely, when strongest lower limb muscle recruitment is needed (Gwin et al., 2011; Seeber et al., 2014, 2015).

To examine the relation between primary motor cortex activity and locomotion in mouse lemurs (*Microcebus murinus*), we introduced two tasks with different biomechanical demands, which consisted of moving on small-diameter substrates oriented horizontally or vertically. Varying substrate orientation modifies body axis orientation and requires adjustments in limb posture (Reghem et al., 2012) and biomechanics (Hesse et al., 2015; Hanna et al., 2017). In horizontal substrates, decreasing diameter induces higher demands for stability, to which primates generally respond by adjusting body posture (high forelimb protraction and elbow flexion; small shoulder height), kinematics (long contact duration, low velocity), and forces (low peak substrate reaction forces; Schmitt, 1999, 2003b; Young et al., 2016). Horizontal and vertical substrates imply distinct functional differentiation of fore- and hindlimbs (Hesse et al., 2015; Hanna et al., 2017). The horizontal condition is characterized by a net-braking role of forelimbs and a net-propulsive role of hindlimbs (Hanna et al., 2017). By contrast, on vertical substrates, fore- and hindlimbs both serve a propulsive role, with greater contribution of hindlimbs, higher compressive loads on hindlimbs and tensile loads on forelimbs (Hanna et al., 2017).

Postural maintenance also varies with substrate orientation in terms of balance challenges and muscle strength required to oppose gravity. These features potentially relate to differences in motor cortex activity, as shown by stronger β power at rest on horizontal than vertical substrates in common marmosets (Tia et al., 2021). Experiments in humans revealed β power increase in frontal, parietal and occipital regions during balance challenges (e.g., platform perturbation, reduced base of support, increased surface compliance; Wittenberg et al., 2017). Bursts of low γ activity also occur in fronto-central regions at the detection of postural instability (Slobounov et al., 2005). Concurrent findings showed that body posture *per se* can tune neuronal discharge in rat posterior parietal and frontal motor cortices (Mimica et al., 2018).

In the current work, we address the question of cortical contribution to locomotor control by examining high β and low γ activity in primary motor cortex of gray mouse lemurs moving on arboreal-like substrates. Mouse lemurs are small strepsirrhines endemic of dry deciduous Malagasy forests (Dammhahn and Kappeler, 2008). They represent our most distantly related primates and possess a prehensile pattern that is phylogenetically conservative (Bishop, 1964; Reghem et al., 2011; Reghem et al., 2012). In this respect, they are highly suitable to reconstruct the mechanisms by which early primates coped with an arboreal environment and developed grasping-related functions (Shapiro et al., 2016). In addition, their small body size facilitates investigation of brain activity in naturalistic contexts.

## Materials and Methods

### Animals

**FIGURE 1:**
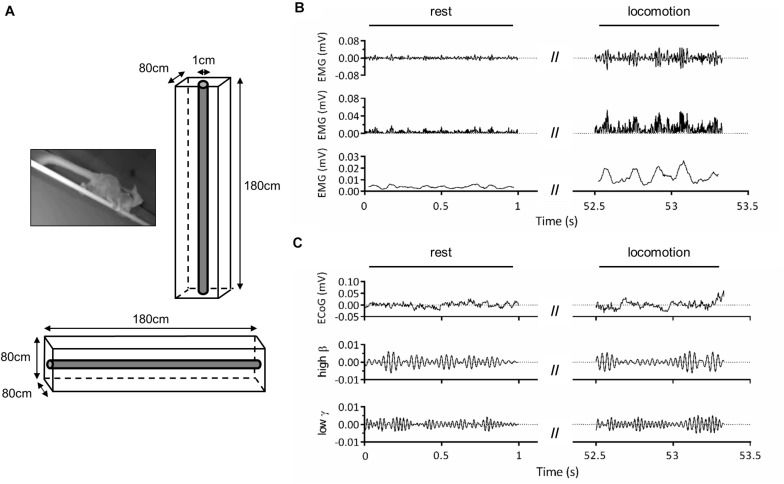
Experimental setup. **(A)** Adult male mouse lemur moving on a 1-cm diameter wooden pole (left panel) and schema of the experimental cage used for vertical and horizontal locomotion tasks (right and bottom panels). **(B)** Representative electromyographic (EMG) activity of the right triceps muscle during locomotion on the horizontal substrate. The first panel represents band-pass (30–500 Hz) filtered EMG signal, the second panel shows the rectified signal, and the third panel, the signal obtained after applying a 50-ms moving average to illustrate cyclic muscle contraction during locomotion. **(C)** Representative electrocorticographic (ECoG) activity of left primary motor cortex during the same trial. The first panel represents the raw signal, the second panel, the high β (25–35 Hz) component and the third panel, the low γ (35–50 Hz) component obtained after band-pass filtering.

Two male gray mouse lemurs (*M. murinus*; ML1, 2.83 years, 83.8 *g*; ML2, 2.92 years, 91.3 *g*; Figure 1A) born and raised in the laboratory colony of UMR 7179 (CNRS/MNHN, Brunoy, France; license approval n° A91.114.1) were used in this study. After surgery, the animals were kept in individual cages enriched with branches and wooden nestboxes. Cages were maintained at a standard temperature of 24–26°C and relative humidity of 55%. The animals were fed with fresh fruits and a laboratory-made porridge of cereals, milk and eggs. Water and food were available *ad libitum*. Animals were tested in summer-like photoperiod (14 h of light/day) and during nocturnal period, which corresponds to their active phase.

### Surgical Procedure

Surgeries were conducted in sterile conditions, under veterinarian supervision. After administration of diazepam (Valium, 1 mg/100 *g*, i.m.) and buprenorphine (0.005 mg/100 *g*, i.m.), anesthesia was induced and maintained by 1–3% isoflurane inhalation. Body temperature was maintained with a heating mat, and the animal’s eyes were protected with ocular gel (Ocry−gel; Laboratoire TVM, Lempdes, France). A small transmitter (PhysioTel F20-EET, 3.9 *g*, 1.9 cc; Data Sciences International, DSI, St. Paul, United States) connected with 2 pairs of electrode wires (silicon elastomer insulated stainless-steel wires, diameter: 0.3 mm) was inserted inside the peritoneal cavity of the animal. One pair of electrode wires was led subcutaneously from the abdomen to the right triceps muscle and was sutured using nonabsorbable polyamide monofilament suture thread. The other pair of electrodes was led subcutaneously to the skull and was implanted epicortically over the left primary motor cortex (coordinates relative to bregma: 2.50 mm anterior and 2.00 mm lateral; 2.50 mm anterior; and 4.00 mm lateral; Bons et al., 1998; Joseph-Mathurin et al., 2010; Nadkarni et al., 2019). After surgery, nociception was minimized by subcutaneous injection of analgesic and anti-inflammatory drug (meloxicam, 0.2 mg/100 *g*).

### Electrophysiological Recordings

Before surgery, the animals were habituated to move on a wooden pole (diameter: 1 cm; length: 180 cm; see also Reghem et al., 2012) fixed at both ends of a wire-mesh cage (180 cm × 80 cm × 80 cm), that could be oriented horizontally or vertically (Figure 1A). The cage was lit by a dim red light. After surgery, the cage was equipped with a wireless telemetry system to record electrocorticographic (ECoG) and electromyographic (EMG) activity during locomotor tasks (Figures 1B,C). The transmitter implanted inside animals’ peritoneal cavity emitted a signal which was received by three antennas (RPC-1, DSI) placed along the cage. These antennas were connected to a hardware interface (matrix MX2, DSI) transferring the signal to the acquisition computer. Signal acquisition was done with Ponemah software (Ponemah Physiology Platform, version 5.10c, DSI) at a sampling rate of 2,000 Hz. Electrode referencing was done by bipolar subtraction. This method, in addition to recording intracranially, close to cortical sources (Darvas et al., 2010; McCrimmon et al., 2018), should minimize contamination of ECoG signal by EMG activity. Locomotor movements were monitored throughout experiments by two infrared IP cameras (30 frames/s; M1145-L, Axis Communications, Lund, Sweden) placed on either side of the cage. Video recording was done with Media Recorder (Noldus Information Technology, Wageningen, Netherlands), synchronized with Ponemah software. Recordings were performed over a period of 2 weeks for ML1 and 1 week for ML2.

### Data Analysis

Using Ponemah software, we annotated and extracted data segments identified as “rest” (absence of movement) and “locomotion” (from start of fore-/hindlimb movement initiating locomotion until end of limb movement terminating locomotion; Figures 1B,C). We excluded from rest all segments within 2 s prior to movement onset to 2 s after the end of locomotion. Intervals with large amplitude artifacts were removed. ECoG and EMG signals provided by all three antennas, which corresponded to different locations of the animal along the pole, were then assembled and notch-filtered at 50 Hz.

We performed two main sets of analyses. The first one involved trials, referring to annotated sequences as defined above. Only the first 500 ms of locomotion trials were retained in order to focus on the initiation part, which more strongly relies on cortical processes (Kiehn, 2016) and to avoid motion noise which occasionally occurs during long sequences of naturalistic locomotion (e.g., when the animal makes abrupt movements). For resting data, 500-ms trials were obtained by segmenting annotated sequences.

The second set of analyses involved locomotor cycles, which were identified based on triceps EMG activity. EMG signal was band-pass filtered (30–500 Hz), rectified, and a moving average with 50-ms window was applied to remove fast signal fluctuations. For each animal and orientation, start and end of a locomotor cycle were defined as instants in which triceps EMG activity exceeded a threshold value defined as one standard deviation above mean resting activity. Individual cycles were controlled on video recordings to exclude data that did not correspond to locomotor movements. For ML1, median cycle duration was 0.169 s on horizontal substrate and 0.253 s on vertical substrate. For ML2, median cycle duration was 0.157 s on horizontal substrate and 0.325 s on vertical substrate. In order to compare locomotor cycles with resting condition, we created “rest cycles” by segmenting annotated rest sequences according to the average of median cycle length across substrate orientations. The total number of trials and cycles per animal and condition is displayed in Table 1.

**TABLE 1:** Number of trials and cycles per animal and condition.

			ML1	ML2
Trials	Horizontal	Rest	144	23
		Locomotion	184	39
		Number of sessions	6	2
	Vertical	Rest	476	65
		Locomotion	210	51
		Number of sessions	6	2
Cycles	Horizontal	Locomotion	43	27
	Vertical	Locomotion	22	13

Electrocorticographic signal was analyzed in high β (25–35 Hz) and low γ (35–50 Hz) bands. The selection of these frequency bands was justified by the channel bandwidth of our implants that covered 1–50 Hz (PhysioTel F20-EET, 3.9 *g*, 1.9 cc; DSI) and by the short duration of locomotor cycles (min ≈ 106 ms) that led us to exclude frequencies below 25 Hz. Previous studies documented the functional significance of high β/low γ frequencies, which display power modulation throughout gait cycle (Gwin et al., 2011; Seeber et al., 2014) and are coupled with muscular activity (Petersen et al., 2012). ECoG signal was transformed into power spectra using a complex Morlet wavelet of three cycles, and average power spectra across high β and low γ frequencies were obtained. All following analyses were applied separately to each frequency band.

We firstly investigated power during rest trials by applying two-way ANOVA with aligned rank transform (ART-ANOVA) with factors of ANIMAL (ML1, ML2) and ORIENTATION (horizontal, vertical). *Post-hoc* pairwise comparisons were performed using Wilcoxon rank-sum test with Bonferroni-Holm correction. We considered *P* < 0.05 as statistically significant. Next, we examined ERD/ERS during locomotion trials. ERD/ERS was defined as a change in power spectrum relative to rest over the same substrate, expressed as a percentage of the resting power. ERD/ERS statistical significance for each condition/animal was assessed by bootstrap tests. For each substrate orientation (horizontal, vertical), we randomly resampled the mean power spectrum of each locomotion trial and created 1,000 bootstrap datasets. These datasets were normalized relative to the mean power spectrum of actual rest trials. A histogram of these bootstrapped ERD/ERS values was then used to test statistical significance. If the 2.5th percentile (ERS) or the 97.5th percentile (ERD) of the distribution of bootstrapped ERD/ERS values was larger or smaller than 0%, respectively, we considered the difference between locomotion and rest as significant. Next, in order to examine modulations of ERD/ERS by experimental conditions, we conducted two-way ART-ANOVA with factors of ANIMAL (ML1, ML2) and ORIENTATION (horizontal, vertical).

In the following set of analyses, we investigated intra-cycle changes in spectrograms. For each condition/animal, ECoG spectrograms of individual cycles were normalized by the average value across duration and cycles. We refer to these changes from average as event-related spectral perturbations (ERSPs; see also Gwin et al., 2011). To statistically assess ERSP modulations throughout gait cycle, each cycle was segmented into two epochs comprising 0–50% (h1) and 50–100% (h2) of cycle length. Conditions were then compared using three-way ART-ANOVA with factors of ANIMAL (ML1, ML2), ORIENTATION (horizontal, vertical), and EPOCH (h1, h2), followed by Wilcoxon pairwise comparisons.

Finally, to verify that triceps EMG activity significantly increased during locomotor cycles and was thus a reliable signal to determine cycle start and end, we compared EMG root-mean-square (RMS) across conditions. Given the low number of locomotor cycles (Table 1), only the first 100 rest cycles were included. For each animal, EMG RMS was calculated and normalized by its maximum value across cycles. We then applied three-way ART-ANOVA with factors of ANIMAL (ML1, ML2), ORIENTATION (horizontal, vertical), and EPOCH (rest, locomotion). To further assess whether the duration of locomotor cycles varied with substrate orientation, we performed a two-way ART-ANOVA on cycle duration with factors of ANIMAL (ML1, ML2) and ORIENTATION (horizontal, vertical). Analyses were performed with Matlab 2020a and the Fieldtrip toolbox (Oostenveld et al., 2011).

## Results

### Power Modulations During Rest and Locomotion

**FIGURE 2:**
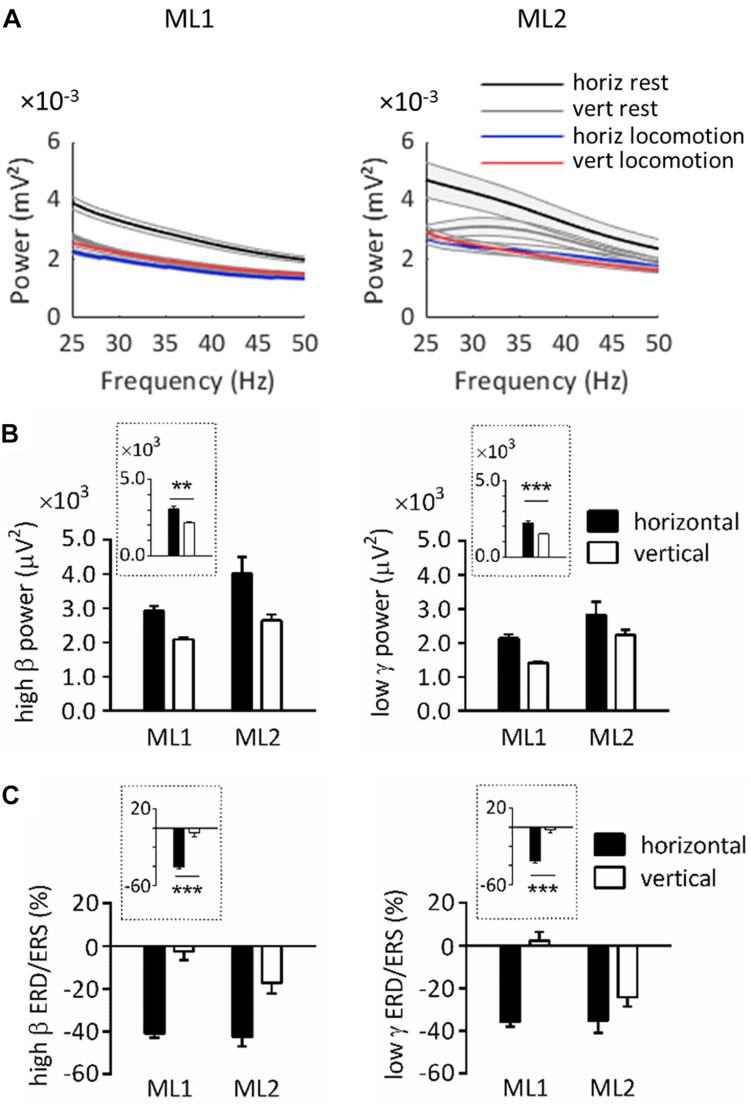
Spectral power over left primary motor cortex in horizontal and vertical conditions. **(A)** Power spectrum obtained for ML1 and ML2 during horizontal/vertical rest and locomotion. The shaded zone represents one standard error of the mean. **(B)** High β and low γ power at rest, across animals and substrate orientations. **(C)** High β and low γ ERD/ERS during locomotion, across animals and substrate orientations. Plots surrounded by a dot line emphasize orientation-related differences averaged across animals. All measurements are expressed as mean ± standard error of the mean (SEM). **p < 0.01 and ***p < 0.001, main effect of orientation as revealed by two-way ANOVAs with aligned rank transform (factors: animal, orientation). horiz, horizontal; vert, vertical.

Figure 2A illustrates the power spectrum in horizontal and vertical conditions for ML1-2. As can be seen, on horizontal substrate, power at rest exceeded power during task in the 25–50 Hz range. For vertical substrate, this was also true for ML2, as well as ML1 in a more restricted interval (≈25–30 Hz). These results agree with typical task-related power variations (Miller et al., 2007; Crone et al., 2011).

Note that power at rest always appeared to be higher in horizontal than vertical condition. In order to statistically assess this effect, we conducted ART-ANOVAs with factors of ANIMAL and ORIENTATION on resting power (Figure 2B). In the high β range, this analysis revealed a main effect of ORIENTATION (*F*_1,704_ = 10.4, *p* = 0.001), which confirmed higher values on horizontal than vertical substrate. A similar effect was obtained in the low γ range (*F*_1,698_ = 12.9, *p* < 0.001). Both analyses also yielded a main effect of ANIMAL (high β, *F*_1,704_ = 9.98, *p* = 0.002; low γ, *F*_1,698_ = 20.4, *p* < 0.001). However, the lack of ANIMAL^∗^ORIENTATION interaction suggests that all lemurs followed the same trends regarding orientation-related power differences (high β, *F*_1,704_ = 1.48, *p* = 0.224; low γ, *F*_1,698_ = 0.022, *p* = 0.882).

Next, we examined ERD/ERS during locomotion. ERD/ERS was calculated by normalizing power during task by power at rest over the same pole orientation. Bootstrap tests indicated that high β ERD was statistically significant in horizontal, but not vertical, condition (*p* < 0.05). Low γ ERD was also significant in horizontal condition, whereas vertical condition induced mixed results, with ERD for ML2 and ERS for ML1.

To better evaluate task-related differences in ERD/ERS, we applied ART-ANOVAs with ANIMAL and ORIENTATION as factors (Figure 2C). This analysis performed on high β ERD showed a main effect of ORIENTATION (*F*_1,480_ = 54.4, *p* < 0.001), but nonsignificant effects of ANIMAL (*F*_1,480_ = 0.972, *p* = 0.325) and ANIMAL × ORIENTATION (*F*_1,480_ = 0.207, *p* = 0.649). This result overall confirmed stronger desynchronization for horizontal than vertical task. Analysis in the low γ range revealed main effects of ANIMAL (*F*_1,480_ = 5.21, *p* = 0.023) and ORIENTATION (*F*_1,480_ = 46.0, *p* < 0.001), but no significant interaction effect (*F*_1,480_ = 3.03, *p* = 0.082). Similar to the high β range, these effects agree with stronger desynchronization for horizontal than vertical task.

### Event-Related Spectral Perturbations During Locomotor Cycle

**FIGURE 3:**
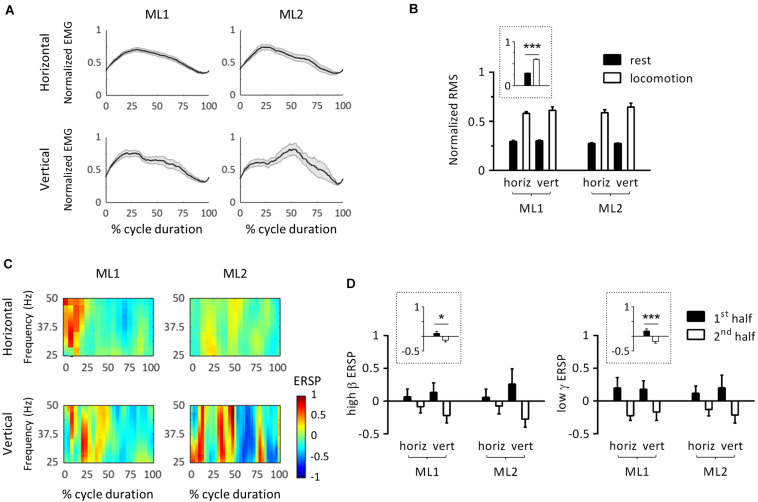
Event-related spectral perturbations (ERSPs) in left primary motor cortex during locomotor cycles. **(A)** Time-course of electromyographic (EMG) activity of the right triceps muscle during locomotor cycles. For each animal, EMG was normalized by maximum root-mean-square (RMS). In this panel, cycle duration was normalized by time-warping individual cycles to median cycle length. The black line represents the average across cycles, and the gray zone, one standard error of the mean. **(B)** RMS of triceps EMG activity across animals, substrate orientations and epochs. For each animal, RMS was normalized by its maximum value. The plot surrounded by a dot line emphasizes epoch-related differences in RMS averaged across animals and orientations. **(C)** ERSP plots showing average changes in spectral power relative to full gait cycle. In this panel, cycle duration was normalized by time-warping individual cycles to median cycle length. **(D)** Average ERSP in high β (left panel) and low γ (right panel) bands across animals, substrate orientations and epochs. Plots surrounded by a dot line emphasize epoch-related differences in ERSP averaged across animals and orientations. *p < 0.05 and ***p < 0.001, main effect of epoch as revealed by three-way ANOVA with aligned-rank transform (factors: animal, orientation, and epoch). All measurements are expressed as mean ± standard error of the mean (SEM). horiz, horizontal; vert, vertical. 1st half and 2nd half refer to the corresponding period of locomotor cycles.

The next set of analyses was conducted to study locomotor cycles that were identified based on triceps muscle activity. Figure 3A depicts the average EMG activity profile for each condition/animal. In this panel, cycle duration was normalized by time-warping individual cycles to median cycle length. Activity was reproducible across locomotor cycles for each task, as evidenced by the relatively small standard error. To verify that EMG activity significantly increased during locomotion compared to rest, we performed an ART-ANOVA with factors of ANIMAL, ORIENTATION and EPOCH (rest, locomotion) on RMS (Figure 3B). This analysis yielded a significant EPOCH effect (*F*_1,481_ = 395, *p* < 0.001) which confirmed higher activity during locomotion than rest. Except for ANIMAL (*F*_1,481_ = 8.77, *p* = 0.003), all other factors were nonsignificant (ORIENTATION, *F*_1,481_ = 2.76, *p* = 0.098; ANIMAL × EPOCH, *F*_1,481_ = 2.10, *p* = 0.148; ANIMAL × ORIENTATION, *F*_1,481_ = 0.766, *p* = 0.382; EPOCH × ORIENTATION, *F*_1,481_ = 1.11, *p* = 0.293; ANIMAL × EPOCH × ORIENTATION, *F*_1,481_ = 0.643, *p* = 0.423).

To further examine whether cycle duration varied between conditions, we conducted an ART-ANOVA with ANIMAL and ORIENTATION as factors. Results showed significant ANIMAL^∗^ORIENTATION interaction (*F*_1,101_ = 9.45, *p* = 0.003) with longer cycle duration on vertical than horizontal substrate for both animals, as revealed by Wilcoxon *post-hoc* tests. We also detected a main effect of ORIENTATION (*F*_1,101_ = 38.6, *p* < 0.001), but no effect of ANIMAL (*F*_1,101_ = 1.47, *p* = 0.229).

In order to study modulations of cortical activity throughout locomotor cycles, we calculated ERSPs by normalizing individual spectrograms by average value across duration and cycles. Figure 3C represents the average ERSP profile per condition/animal. For this panel, cycle duration was normalized by time-warping individual cycles to median cycle length. Modulations of spectral power generally consisted of positive values in the first half cycle (i.e., increase relative to mean power), and negative values in the second half cycle (i.e., decrease relative to mean power). This pattern, which covered the 25–50 Hz range, was similar across animals and orientations. To statistically evaluate this effect, we calculated mean ERSP in the first and second half cycle and applied ART-ANOVAs with factors of ANIMAL, ORIENTATION and EPOCH (h1, h2). Separate analyses were conducted for high β and low γ bands. In both cases, results confirmed a main effect of EPOCH (high β: *F*_1,178_ = 4.29, *p* = 0.040; low γ: *F*_1,180_ = 12.9, *p* < 0.001) with higher values in the first than second half cycle (Figure 3D). No other main effect or interaction was detected in the high β band (ANIMAL, *F*_1,178_ = 0.026, *p* = 0.872; ORIENTATION, *F*_1,178_ = 0.000, *p* = 0.994; ANIMAL × ORIENTATION, *F*_1,178_ = 0.006, *p* = 0.941; ANIMAL × EPOCH, *F*_1,178_ = 0.003, *p* = 0.960; ORIENTATION × EPOCH, *F*_1,178_ = 1.70, *p* = 0.195; and ANIMAL × ORIENTATION × EPOCH, *F*_1,178_ = 0.022, *p* = 0.883), or in the low γ band (ANIMAL, *F*_1,180_ = 0.225, *p* = 0.636; ORIENTATION, *F*_1,180_ = 0.073, *p* = 0.787; ANIMAL × ORIENTATION, *F*_1,180_ = 0.155, *p* = 0.694; ANIMAL × EPOCH, *F*_1,180_ = 0.149, *p* = 0.700; ORIENTATION × EPOCH, *F*_1,180_ = 0.166, *p* = 0.684; and ANIMAL × ORIENTATION × EPOCH, *F*_1,180_ = 0.022, *p* = 0.881). In particular, the lack of ANIMAL × EPOCH and ANIMAL × ORIENTATION × EPOCH interactions confirm that all animals followed the same pattern regarding EPOCH-related ERSP variations. Of note, substrate orientation did not appear to significantly modulate ERSPs.

## Discussion

In this study, we examined oscillatory dynamics in mouse lemur primary motor cortex during locomotion on small-diameter horizontal/vertical substrates. Our results highlight significant effects of substrate orientation on high β and low γ power at rest (i.e., postural maintenance) and on ERD during locomotion. Furthermore, we bring evidence of intra-cycle modulation of cortical activity, with stronger power in the first than second half of locomotor cycles. In the following sections, we discuss these results and propose possible underlying mechanisms.

### Substrate Orientation Modulates Power at Rest

Stronger power at rest on horizontal than vertical substrate echoes earlier findings on β oscillations (e.g., common marmosets; Tia et al., 2021) and could relate to different body posture and muscular activity required to oppose gravity (Hesse et al., 2015; Hanna et al., 2017). It could further reflect stronger balance challenges on horizontal than vertical substrates (Wittenberg et al., 2017). Evidence in macaques and humans suggests that β oscillations relate to the control of stable posture and promote tonic motor activity at the expense of voluntary movement (Spinks et al., 2008; Kilavik et al., 2013; Zaepffel et al., 2013). Since cortical oscillations reflect net dendritic synaptic currents, different power indicates that populations of neurons are active to different extents depending on body posture (Spinks et al., 2008). A possible functional hypothesis for the role of β oscillations states that they could facilitate the upscaling of somatosensory responsiveness, in parallel with increased sensorimotor communication during stable posture, resulting in an updating of the internal representation of current body status (Kilavik et al., 2013). In contrast with β oscillations, low γ oscillations were less often linked to postural maintenance, although premotor and primary motor cortex show some degree of selectivity for stable hand configurations in this frequency range (Spinks et al., 2008). Low γ activity could reflect neural processes for balance monitoring and triggering of stabilization mechanisms, as suggested by bursts of activity in fronto-central regions at the detection of postural instability (Slobounov et al., 2005).

To our knowledge, few studies showed differences in cortical resting activity related to whole-body posture in non-human primates (but see Tia et al., 2021). By contrast, in humans, previous work addressing this question showed significant enhancement of high frequencies (20–65 Hz) and reduction of low frequencies (<4 Hz) in upright/inclined compared to supine posture (Spironelli et al., 2016; Thibault and Raz, 2016; Spironelli and Angrilli, 2017). This effect was seen as a consequence of the redistribution of gravitational loads. In humans, supine compared to seated/inclined postures enhances blood flow toward the head and mimics increased blood pressure, which stimulates arterial and cardiopulmonary baroreceptors, leading to a decrease in sympathetic nervous system activity and an enhancement of cortical inhibition (Spironelli et al., 2016; Thibault and Raz, 2016). Possible cross-species differences in cardiovascular and sympathetic nervous responses limit the generalization of this interpretation to quadrupedal primates. An alternative, context-dependent, hypothesis proposes that possible interactions between the current posture and the surrounding environment could modulate brain activity (Thibault and Raz, 2016). This view is coherent with the fact that motor plans depend on ongoing limb configuration (de Lange et al., 2006). However, in the present work, the large variety of active postures in arboreal environments makes it difficult to relate substrate orientation with different levels of cortical excitability.

### Substrate Orientation Modulates ERD During Locomotion

Our results indicate that high β and low γ oscillations were suppressed during locomotion on horizontal substrate as compared to rest. There is a general consensus on the fact that β oscillations are characterized by amplitude decrease during movement in relatively broad sensorimotor areas (Pfurtscheller et al., 2003; Miller et al., 2007). In humans, during active walking, β oscillations are suppressed in central sensorimotor areas as compared to upright standing (Seeber et al., 2014). The same is true for common marmosets during locomotion on horizontal/vertical poles as compared to rest (Tia et al., 2021). β ERD is considered to reflect movement-related enhancement in cortical excitability (Seeber et al., 2014, 2015). In macaque motor cortex, β oscillations negatively covary with neuron firing rate during movement, such that small and desynchronized β activity associates with peak neuronal discharge (Spinks et al., 2008). In general, low frequency (8–32 Hz) power changes are presumed to arise in cortical regions collectively regulated by central structures such as the thalamus and basal ganglia (Miller et al., 2007). In contrast with β, reports on low γ ERD/ERS vary depending on the task and methodology (Szurhaj et al., 2003; Omlor et al., 2007; Babiloni et al., 2016), which may be due to the variability of frequencies over which power suppression (α, β) and power increase (high γ) occur and extend into the low γ range (Crone et al., 2011). Low γ oscillations reflect small neuronal populations involved in local processing along a distributed cortical circuit (Babiloni et al., 2016). They are involved in rapid integration of sensory signals and production of the motor command (Omlor et al., 2007).

Intriguingly, we found no β ERD and inconsistent γ ERD/ERS in the vertical condition, contrary to the horizontal one. These results could stem from discrepancies between our resting condition and standard baseline conditions of other studies. Here, we required animals to actively maintain whole-body posture by applying grip forces onto the substrate to oppose gravity. By contrast, resting condition in humans generally consists of upright standing on a flat surface (Seeber et al., 2014, 2015). Differences in resting power could entail differences in ERD/ERS between conditions, provided that power during locomotion does not greatly differ. An alternative hypothesis would be that the presence/absence of ERD/ERS reflects processes of locomotor adaptation to substrate properties. β ERD in sensorimotor regions generally displays little specificity to spatial and temporal components of the task, but movement-related power decrease is somatotopically organized following the classic homunculus (Miller et al., 2007; Kilavik et al., 2013). Similar somatotopic organization of low γ power variations was documented (Crone et al., 1998; Szurhaj et al., 2005). Relating this to our results, we can presume that different distribution of muscle activity at rest/during task on horizontal/vertical substrates could partly account for observed effects. Recent work further highlighted possible involvement of β and γ oscillations in gait stability requirements, as shown by reduced sensorimotor power in human subjects while walking on a balance beam compared to a treadmill (Sipp et al., 2013). Thus, differences in ERD depending on substrate orientation could as well reflect different balance requirements. Quantifying behavioral performance (e.g., kinematic measurements, gait classification) could provide an indicator of task difficulty and clarify this hypothesis. Regarding this question, previous work in mouse lemurs reported no difference in preferred gait when using small diameters of various orientations (horizontal, 30°incline/decline), and no significant modulation of speed by diameter or incline (≈1.5–2 m/s; Shapiro et al., 2016). Thus, mouse lemurs seem able to adapt their locomotor strategy to reach similar performance on different types of substrates.

Modulation of ERD by substrate orientation contrasts with previous work in common marmosets (Tia et al., 2021), where authors reported rare effects of orientation as opposed to sharper effects of gait in the β range. As mentioned earlier, gait, and more generally kinematics, were not evaluated in this work and could be a source of variability between conditions. Besides, other factors could account for discrepancies between these two studies, like different frequency ranges (mouse lemur, 25–50 Hz; marmoset, 16–35 Hz; Tia et al., 2021) and cross-species differences in anatomy and physiology derived from specializations to distinct ecological niches (Schmitt, 2003a; Dammhahn and Kappeler, 2008).

### Intra-cycle Modulation of Motor Cortex Activity

A major finding of this study is the intra-cycle modulation of cortical activity in mouse lemurs, which echoes previous work on cortical coupling to gait cycle phase in humans (Gwin et al., 2011; Seeber et al., 2014, 2015). The novelty of our results lies in the arboreal, quadrupedal sequence gait of mouse lemurs as opposed to human bipedal stepping. Our cycle start coincides with an increase in right triceps muscle activity, which is typically observed at the beginning of stance phase (e.g., in macaques; Courtine et al., 2005). Assuming that mouse lemurs used an asymmetrical gait, as is most frequently observed in this species (Shapiro et al., 2016), left and right forelimbs should be temporally paired, like left and right hindlimbs. In the case of transverse gallop, fore- and hind leading limbs should be on the same side of the body (e.g., left hindlimb – right hindlimb – left forelimb – right forelimb). By contrast, if the animals used a symmetrical sequence gait such as walk or amble, a forelimb would be temporally paired with a hindlimb, generally the contralateral one. Although it is difficult to correlate cortical activity with a specific sequence of limb movements, our results bring clear evidence of high β/low γ power modulation throughout gait cycle, presumably with higher values during contralateral forelimb stance and lower values during swing.

In humans, intra-stride modulation of electrocortical activity is visible in multiple areas and encompasses several frequency bands (Gwin et al., 2011; Seeber et al., 2014, 2015). β power increase in sensorimotor cortex is most pronounced at the end of contralateral limb stance when maximum lower limb muscle force is required for push-off, which suggests that cortical power in certain frequency bands could index muscle recruitment (Gwin et al., 2011). In rats, neuronal population responses in hindlimb motor cortex are synchronized with spatiotemporal activity of motoneurons and closely parallel the modulation of hindlimb kinematics and muscle activity during locomotion (Digiovanna et al., 2016). During precision walking, temporal tuning of muscle synergies is reflected in a shift of peak firing rate of neuronal population responses, whereas during stair climbing, increased muscle activity is reflected in a global enhancement of neuron firing rates. These observations lead to the idea that the modulation of cortical activity throughout gait cycle could reflect the supervision of downstream circuits involved in limb movement production.

Surprisingly, we did not detect any orientation-related effect on intra-cycle modulation of cortical activity. This was unexpected, considering differences in biomechanical contributions of fore- and hindlimbs on horizontal/vertical substrates (Reghem et al., 2012; Hesse et al., 2015; Hanna et al., 2017) and potential differences in kinematics (e.g., cycle duration) that could impact motor control processes. The small size of our dataset could be a limitation to effect detection. The question of orientation effects and, more generally, modulation of electrocortical activity during gait cycle would deserve deeper investigation and complementary measurements of limb biomechanics along with precise somatotopy of recorded cortical regions.

### Functional Considerations on High β and Low γ Ranges

This study focused on cortical oscillations in the 25–50 Hz range, which spans some β frequencies where ERD is classically reported (18–30 Hz) and high β/low γ frequencies where intra-cycle modulations were described (24–40 Hz) in human locomotion (Seeber et al., 2014, 2015). Although in humans, β and low γ modulations partly overlap in their frequency range and cortical location, they exhibit different spectral peaks, which suggests that they could subtend different mechanisms. β ERD is considered to reflect movement-related enhancement in cortical excitability, whereas low γ modulation would represent sensorimotor processing linked to motion sequences during gait. Our results, along with previous findings, imply that intra-cycle amplitude modulation is compatible with a certain ERD level. In humans, the 24–40 Hz range is further coupled to higher γ oscillations (70–90 Hz), with both ranges varying conversely to each other in relation to the gait cycle (Seeber et al., 2015). The functional meaning of this phenomenon remains to be fully elucidated.

A potential issue, already raised in the literature, is that of possible differences in frequency ranges ensuring cortical control of muscle activity during static posture *vs* movement. This idea is supported by recent findings on corticomuscular coherence between primary motor cortex and *tibialis anterior* muscle in humans, where authors reported peak coupling in the 15–30 Hz range during static contraction, but in the 24–40 Hz range during the swing phase of walking (Petersen et al., 2012). A similar drift in frequencies was previously described for upper limb movements (Omlor et al., 2007) and could signify that different functional networks are at work during isometric (e.g., postural maintenance) *vs* phasic movements (e.g., locomotion). Although no such effect emerged in our study, targeting lower frequencies (e.g., 15–25 Hz) and exploiting complementary techniques to link cortical and muscular activity could help to better address this question.

## Concluding Remarks

In this study, we investigated how high β and low γ oscillations in mouse lemur primary motor cortex relate to postural maintenance and locomotion on different substrate orientations. Key originalities of this work were to exploit an experimental design approaching naturalistic conditions, i.e., modeling the fine-branch niche (e.g., small substrate diameter, various orientations), and to examine an animal model representative of ancestral primate features, thereby providing a means to extrapolate how early primates might have responded to variable substrates (Shapiro et al., 2016). Our findings bring evidence that mouse lemurs adjust cortical activity to substrate orientation during static posture and locomotion, and modulate this activity throughout locomotor cycle. This provides one of the first descriptions of cortical mechanisms involved in mouse lemur locomotion. The idea that specific processes are solicited during arboreal locomotion adds to existing knowledge on primate locomotor evolution, derived from studies on biomechanical benefits and challenges encountered by small animals on arboreal substrates. Our study also yields perspectives in the biomedical field related to locomotor functions and impairments. Research on healthy and pathological aging in mouse lemurs demonstrated that this animal is a unique model to study age-dependent changes in sensory, motor and cognitive functions (Languille et al., 2012). Mouse lemurs display several features in common with human aging, such as a decrease in balance and motor capacities. Thus, this study could set the basis for deeper investigation of locomotor processes and serve as a reference when evaluating their degradation.

## Data Availability Statement

The raw data supporting the conclusions of this article will be made available by the authors, without undue reservation.

## Ethics Statement

The animal study was reviewed and approved by the local ethics committee “Comité d’éthique Cuvier” (authorization APAFIS#2083-2015090311335786).

## Author Contributions

BT and FP contributed to conception and design of the study. FP performed the surgeries. BT conducted the experiments, analyzed the data, and wrote the first draft of the manuscript. Both authors contributed to manuscript revision, read and approved the submitted version.

## Conflict of Interest

The authors declare that the research was conducted in the absence of any commercial or financial relationships that could be construed as a potential conflict of interest.
